# Nurturing creativity in comfort; Gently pushing the boundaries within a zone of safety

**DOI:** 10.1371/journal.pone.0313553

**Published:** 2026-03-10

**Authors:** Carlota Torrents, Sílvia Garcías de Ves, Matthew Bourke, Veronique Richard

**Affiliations:** 1 Insitut Nacional d’Educació Física de Catalunya (INEFC), University of Lleida, Lleida, Spain; 2 School of Human Movement and Nutrition Sciences, The University of Queensland, St Lucia, Australia; 3 Health and Wellbeing Centre for Research Innovation, San Francisco, California, United States of America; RMIT University, AUSTRALIA

## Abstract

In today’s rapidly evolving world, fostering creativity within educational settings is essential to prepare individuals for complex global challenges. This study explores the dynamic interplay between individuals and their environments in nurturing creative potential. The research focuses on the influence of participants’ experiences of challenge, comfort, destabilization, support, connection, engagement, and emotions on developing students’ creative potential throughout a creative dance intervention. Ninety-three university students (62 men and 35 women; Mean age: 20.5) participated in 36 creative dance classes over four months. Creative self-efficacy, tolerance for ambiguity, emotional creativity, and ideational behaviors were assessed before and after the intervention. After every class, a brief questionnaire assessing students’ experience of creative dance was filled out. A two-step approach, firstly estimating a mixed effect location scale model and secondly following a simpler linear regression model, was applied to assess how variations in the students’ experience of the creative dance program impact creativity-related variables. Contrary to the common belief that discomfort and stepping out of one’s comfort zone are necessary for creative growth, the findings suggest a more nuanced relationship. While challenges and destabilizing activities can stimulate creativity by preventing routine responses, the study found that comfort, support, and positive emotional experiences were significantly associated with creative self-efficacy and emotional creativity. Social support and connection emerged as critical factors in nurturing creativity. Environments characterized by psychological safety, where individuals feel secure to take risks without fear of negative consequences, seemed to be associated with creative engagement. The formation of group synergies through shared goals and mutual trust were associated with innovative outcomes and individual creative development. The study also highlighted the importance of consistent engagement and positive emotional experiences in fostering tolerance for ambiguity and ideational behaviors. While consistent support and engagement were linked to a greater ability to tolerate ambiguity, variability in peer connection and emotional responses during collaborative processes seemed to be associated with creative ideation. In conclusion, the research emphasizes the significance of person-environment interactions in creative development interventions. Supportive and comfortable environments that foster positive connections and emotional well-being were more consistently associated with favourable creativity-related outcomes than environments centred primarily on challenge. Integrating creativity and emotional awareness into educational practices, particularly in movement-based activities like dance and physical education, can unlock the full potential of these disciplines and better prepare individuals for the complexities of the modern world.

## Introduction

Cultivating creativity, traditionally defined as producing novel and appropriate work within a given context [1], is paramount in an era characterized by rapid technological advancements, multifaceted global challenges, and evolving socio-cultural landscapes [2,3]. Although the exact definition of creativity is subject to ongoing debate [e.g., 4, 5], there is a consensus among scholars that fostering one’s creative potential requires developing a distinctive combination of cognitive, affective, and technical/physical skills [6,7]. More importantly, Studies of Change and Development [SCDs; 8] posit that these creative potentialities interact continually with environmental constraints shaped by cultural, social, and material influences [9–11]. Hence, focusing solely on developing individuals’ skills is insufficient to foster a positive trajectory in creative change and development. Interventions must also consider how people interact with their environment, aligning with sociocultural perspectives that view creativity deeply embedded in its context [12]. Creativity emerges through continuous interactions between individuals and their social, material, and cultural surroundings, implying that the same activity may lead to different developmental outcomes depending on how it is experienced.

Despite this shift, most intervention studies aimed at enhancing creativity continue to evaluate outcomes while treating the learning environment as a background condition rather than an explanatory variable. Environmental features such as perceived challenge, comfort, social support, emotional safety, and engagement are often discussed *post hoc* but are rarely directly measured or modelled longitudinally. As a result, limited empirical evidence exists on how participants’ lived experiences during creativity-supportive interventions contribute to changes in creativity-related skills.

Movement-based and embodied practices offer a particularly relevant context to examine these dynamics. Creative dance, in particular, can evoke a wide range of experiential states: it may be perceived as challenging, destabilising, emotionally intense, socially exposing, but also as enjoyable, supportive, and connecting. These characteristics make creative dance a promising context for studying how person–environment interactions shape creative development over time.

The present study addresses this gap by examining how students’ experiences during a semester-long creative dance course relate to changes in creativity-related outcomes. Rather than focusing solely on the intervention content, we explicitly model participants’ subjective experiences and examine how both their average levels and session-to-session variability are associated with creative development.

Specifically, we investigate creativity-related skills identified as key facilitators of creative potential, including creative self-efficacy, tolerance for ambiguity, emotional creativity, and ideational behaviours. By adopting a longitudinal design with repeated assessments of in-class experiences, this study aims to contribute to a more nuanced understanding of how creative potential develops through sustained engagement in embodied, socially situated learning environments.

Accordingly, the study addresses the following research questions:

1. Do experiences of challenge, comfort, destabilization, social support, engagement, and emotions differ between creative movement classes and theoretical or presentation-based classes?
2. How do students’ experiences of challenge, comfort, destabilization, social support, and connection during creative dance classes relate to changes in creativity-related skills across the intervention?
3. How do levels of engagement and emotional experiences during the intervention influence creativity-related outcomes?

### Literature review

#### Socio-cultural and ecological perspectives.

Vygotsky [12] argued that creativity emerges through social interactions and cultural tools, such as language and symbols, which mediate human activity. Building on this, Sawyer [13] highlighted the collaborative and distributed nature of creativity, suggesting that group dynamics, shared knowledge, and collective problem-solving significantly shape creative outcomes. These insights are particularly relevant in educational settings, where creativity can be fostered through collaborative activities and environments that encourage dialogue, mutual support, and the exchange of diverse perspectives. This aligns with Glăveanu [14] five A’s framework, which proposes a socio-cultural perspective of creativity underscoring the interplay between the actor, the audience, the action, the affordance (i.e., possibility for actions), and the artefact. According to such socio-cultural perspective, the ‘creative act’ is inherently relational, involving interactions between the creative actor(s), their audience, and the multiple possibilities afforded by the sociocultural context in which they operate [15]. This perspective challenges traditional, individualistic, and outcome-oriented views of creativity by highlighting the importance of community, tradition, and shared meaning-making in the creative process [9]. Given these socio-cultural perspectives, this study conceptualizes creativity as a process that both explores and expands possibilities for self, groups, and society [16].

Despite the important contributions of socio-cultural approaches to the field of creativity research [9], few intervention studies that aim to foster specific dimensions of an individual’s creative potential have considered person-environment interactions as explanatory variables for creative change and development. This is unsurprising, as creativity research often stems from the field of psychology, which has historically prioritized individual and cognitive factors over environmental influences [17]. Yet, according to the seminal work of Eleanor J. Gibson [18], learning occurs when individuals (also called ‘perceivers’) actively explore their environment to discover what it affords. Through this perception-action process, individuals become more attuned to environmental features. The environment can thus be conceptualized as a rich landscape of affordances available for one to perceive and actively engage with [19]. While the physical environment plays a crucial role in perceptual learning, the socio-cultural environment is equally significant. For example, if an individual perceives a risk of being judged negatively for expressing unconventional ideas, they may choose to withhold their ideas, thereby limiting creative expression. In other words, how individuals interact with an environment can shape their potential for creative growth.

### Challenge, (dis)comfort, and disequilibrium?

One factor potentially complexifying the study of person-environment interactions on creative development is the multitude of paradoxes characterizing creative environments. Freedom versus constraints [11], structure versus chaos [20], or challenge versus comfort, it remains ambiguous which environment most effectively fosters creative development.

The concept of challenge is often associated with pushing individuals beyond their comfort zones, fostering growth, and unlocking creative potential. Social networks are full of advertisements encouraging people to engage with activities out of their “comfort zone” to be more innovative. Although this concept also transcends popular psychology literature [e.g., 21], scientific evidence examining the impact of “stepping out of one’s comfort zone” is scarce [22].

Defined broadly as undertaking behaviors that individuals find difficult [22], venturing out of one’s comfort zone entails the exploration of unknown, unfamiliar, and unpredictable “spaces”. Nadler [23] highlighted how outdoor adventure education’s challenges foster safe “edgework”, stretching personal boundaries to bolster resilience, self-confidence, and self-awareness. Extending these insights, Van Gelderen [24] introduced the concept of a “learning zone” between comfort and panic, revealing through research that the essence of this zone lies in surprise rather than discomfort. In this vein, the seminal work of Csikszentmihalyi [25] on “flow” argues that the optimal creative state often resides where challenges and skills are delicately balanced. This line of work suggests safe, surprising, and balanced challenges supporting learning and creativity. This beckons the question of why discomfort remains a prevalent theme. Is comfort antithetical to solving complex problems or acquiring new skills, or does it play a role in enhancing creativity?

Kaufman and Beghetto [26] propose a nuanced view, suggesting that individual differences and contextual nuances influence the relationship between discomfort and creativity. Even highly creative individuals may seek strategies or routines to kindle inspiration, not necessarily stepping outside their comfort zones but finding their ideal conditions for creativity. These personal conditions, tailored to individual preferences and personality traits, may nudge them beyond usual responses without neglecting comfort.

Less frequently discussed in popular literature than the concept of the “comfort zone”, the experience of disequilibrium may explain, at least partially, the link between engaging in challenging activities and creative potential fulfilment. For instance, intentionally setting constraints is a powerful strategy to enhance creativity [27]. By preventing routine responses, specific task constraints release new degrees of freedom and delineate exploration areas, allowing for the emergence of novel possibilities. Although this approach may temporarily disrupt equilibrium, it is essential for learning, as adopting new behaviors requires disturbances in stable states. When constraints disturb the system, it enters a state of disequilibrium, prompting an exploration of cognitive, emotional, physical, and social solutions to reorganize into more functional states [10,28,29]. These constraints shape behaviors without undermining the joy of creation or the creators’ well-being.

### Social support and connection

While the impact of challenging, uncomfortable, and destabilizing activities on creative development remains uncertain, the literature recognizes the importance of social support and connection as pivotal in nurturing creativity. Specifically, research suggests that to foster creativity, educators should create an environment where ideas are valued, mistakes are seen as essential to the learning journey, and relationships with students are cultivated positively [30–32]. Building on this, the concept of psychological safety –an individual’s perception of the consequences of taking interpersonal risks [33] – emerges as crucial. Evidence indicates that psychological safety is vital in encouraging participation in creative endeavours by ensuring individuals feel protected from embarrassment or ridicule for their original ideas [34,35]. In such an environment, individuals must feel secure and respected within the group to fully engage and contribute to creative processes.

This does not mean that the formation of groups should solely rely on members sharing similar behaviors or beliefs, promoting a “relaxed process”. On the contrary, the configuration of groups with diverse backgrounds, talents, and expertise can support creative growth [36]. This diversity encourages cooperation and competition among ideas, favoring the emergence of synergies between the group members. Synergies are characterized by the coordination between the different members who interact in a coordinated and flexible manner [27]. Facilitating effective synergies is essential in group interventions aiming at fostering creative potential. This involves setting common goals that align with each member’s motivations and interests and promoting mutual trust and respect to navigate the tensions or disagreements that might arise from collective creative endeavours [37,38]. When these conditions are respected, it is usually observed that team outcomes are more creative, which, in turn, benefits each member’s creative development [39]. Therefore, fostering a sense of social support and connection among group members is a critical environmental condition in creative interventions.

### Engagement and emotional experience

As previously mentioned, individuals may be drawn to or deterred from engaging with certain environments. Consequently, to examine the impact of person-environment interactions on the effectiveness of creative-enhancement interventions, it is crucial to consider participants’ engagement with creative activities and the emotional responses they evoke. For instance, integrating creative movement activities into the curriculum initially triggered emotional challenges in higher education, leading to some students’ refusal to participate [40]. Romero-Martín & Caballero-Julia [41] suggest that fear, embarrassment, expectations, and perceptions of low ability can inhibit movement, increasing the likelihood of disengaging with creative movement practices. Similarly, Kiknadze & Leasy [22] found that when faced with “stepping out of their comfort zone”, the apprehension of negative feelings was associated with disengagement behaviors. In contrast, the anticipation of positive feelings leads to approaching behaviors. For cognitive, affective, social, and physical changes to emerge from any person-environment interaction, participants must first decide to engage with the environment. Therefore, ensuring that creativity-supportive interventions evoke positive feelings is critical.

### The current study

Dancing creatively can be challenging, uncomfortable, and destabilizing. Yet, it can also unite people in a moment of collective effervescence, evoking feelings of connection and profound joy. The experience largely depends on how individuals engage with the activity. These contrasting characteristics position creative dance as an ideal approach for exploring how interactions between individuals and their environments shape outcomes from creativity-supportive activities.

The study first aims to examine the overall experience evoked by dancing creatively compared to listening to theory or performing a dance. Then, it delves explicitly into how college students’ experiences of challenge, comfort, destabilization, support, connection, engagement, and emotions—throughout a 15-week creative dance course—contribute to the development of creativity-related skills, including creative self-efficacy, tolerance for ambiguity, emotional creativity, and ideational behaviors. These psychological skills are recognised as important facilitators of the development and expression of creative potential [42–45].

We hypothesize that

H1. Students will report significantly different experiential profiles (challenge, comfort, destabilization, support, engagement, and emotions) in creative movement classes compared to theoretical and presentation-based classes.

H2. Higher perceived comfort, support, connection, and destabilization during creative dance classes will be positively associated with improvements in creativity-related outcomes (creative self-efficacy, tolerance for ambiguity, emotional creativity, ideational behaviors).

H3. Greater engagement and more positive emotional experiences during the intervention will be associated with greater gains in creativity-related outcomes.

## Methods

### Participants

One hundred twenty-three students enrolled in the first year of a Physical Activity and Sport Sciences Degree at the National Institute of Physical Education of Catalonia (University of Lleida) were invited to participate. The recruitment period started on the 07/02/2022 and ended on the 21/02/2022. All participants signed a written informed consent. The students were split into four groups of 25–32 students through the regular university scheduling process. While participation in the creative dance course was mandatory as part of the degree requirements, participating in the study was voluntary. To respect confidentiality and ensure that the study’s results would not impact academic evaluations, a process of blinding the names of the participants was followed. No prior power analysis was conducted since the sample size was determined by the number of students enrolled in the course and could not be modified. Nonetheless, following conventional power benchmarks [46], a sample of approximately 90–100 participants is typically sufficient to detect medium-sized effects (f² ≈ .15) in regression-based analyses.

Of the 123 students, 23 had missing pre- or post-intervention data for study outcomes and were removed from the analysis. Three participants did not comply sufficiently with weekly questionnaires (i.e., completed less than one-third of the weekly questionnaires) and were excluded from the study. Therefore, a total of 97 participants were included in the analysis. Of these 97 participants, 62 (63.9%) were men and 35 were women (36.08%). Participants were 18–35 years old (M = 20.50, SD = 2.68). Most participants were Caucasian (n = 88, 90.7%), from Spain (n = 83, 85.6%), and had no experience with creative movement (n = 77, 79.4%).

The study was approved by the Ethics Committee of the Sport Catalan Government Clinical Research (004/CEICGC/2022) and complied with relevant guidelines and the principles of the Declaration of Helsinki. The study design and data are registered on the Open Science Framework (OSF) with the following DOI: https://doi.org/10.17605/OSF.IO/R2AVM.

### Design and procedure

A quasi-experimental longitudinal design with repeated measures was used to examine the associations between students’ experiences during classes (independent variables) and changes in the study variables (dependent variables). All participants were asked to fill out a demographic form on the first day of the intervention. To examine changes in dependent variables, students were also asked to fill out a series of scales assessing students’ creative self-efficacy, tolerance to ambiguity, emotional creativity, and ideation behaviors. The same scales were administered again at the end of the intervention (36th session). To explore how students’ experience of the creative dance classes influences changes in dependent variables, students were required to fill out a class experience questionnaire at the end of each creative dance session. All scales and questionnaires were completed online during class time.

### Measures

#### Pre and post-intervention measures.

*Creative self-efficacy in movement:* Because there is no all-purpose measure of self-efficacy and the “one measure fits all” approach yields a poor explanatory and predictive value, Bandura [47] designed a guide for constructing tailored self-efficacy scales. These guidelines were followed to create a scale measuring creative self-efficacy tailored to the task demands of the movement intervention implemented in this study. First, because “perceived self-efficacy is a judgment of capability to execute given types of performances” (36, p. 309), the initial instruction asked participants to “rate how confident are you that you can effectively…”. This is also in line with Beghetto & Karwowski [43] recommendations to design measures of creative self-efficacy. Items were then constructed around the key performance features of creative dance. Specifically, two experts (first and last authors) detailed the abilities targeted by creative dance. Ten items were created around participants’ abilities to move in original ways, express their thoughts and emotions through movement, use the environment to move creatively, and create a sequence of movements. Participants rated the strength of their efficacy beliefs on a 100-point scale as recommended [43,47]. The scale was also inspired by prior work on creative self-efficacy [e.g., 48]. Internal consistency was considered excellent at all three time points (Cronbach’s α = 0.949 to 0.965).

#### *The Multiple Stimulus Types Ambiguity Tolerance Scale (MSTAT-II;* [49]).

The MSTAT-II is a 13-item self-report scale asking participants to rate their level of agreement with statements describing behavioral responses to ambiguous situations (i.e., novel, complex, insoluble, and ambiguous in general) on a Likert scale ranging from 1 (strongly disagree) to 5 (strongly agree). The Spanish version has been tested for validity and reliability, revealing very high internal consistency and high temporal stability. Results confirmed that the MSTAT-II translated into Spanish is a valid measure of ambiguity tolerance [50]. Internal consistency was considered acceptable or good at all three-time points (Cronbach’s α = 0.783 to 0.827).

#### *Shortened Spanish version of the Emotional Creativity Inventory (ECI-S;* [51].

The ECI-S consists of a 17-item self-report questionnaire that provides information about emotional preparedness (i.e., understanding emotions’ causes/effects), novelty (i.e., the ability to experience unusual emotions), and effectiveness/authenticity (i.e., expressing emotions genuinely and honestly). Participants were asked to assess their ability to experience and express emotions on a 6-point Likert scale ranging from 1 (strongly disagree) to 6 (strongly agree). The Spanish version showed adequate internal consistency and temporal stability and confirmed the three-factor structure of the original scale. Internal consistency for the preparedness (Cronbach’s α = 0.706 to 0.759), novelty (Cronbach’s α = 0.706 to 0.783), and effectiveness/authenticity (Cronbach’s α = 0.719 to 0.768) subscales were considered acceptable at all three-time points.

#### *Runco Ideation Behaviour Scale (RIBS;* [52]).

RIBS consists of 23 items, of which participants must indicate the frequency of occurrence of each ideational behavior on a scale ranging from 0 (never) to 4 (very often) [44]. The Spanish version confirmed the 2-factor model and showed good discrimination and satisfactory reliability [53]. The first factor includes positive statements about ideation behaviors (e.g., “I like to play around with ideas for the fun of it”), while the second factor depicts negative ones (e.g., “Sometimes I get so interested in a new idea that I forget about other things that I should be doing”). Internal consistency for the total scale was considered good to excellent at all three time points (Cronbach’s α = 0.883 to 0.912), whereas internal consistency for the first factor was considered good at all three time points (Cronbach’s α = 0.863 to 0.898), and the second factor was considered acceptable at all three time points (Cronbach’s α = 0.747 to 0.786).

#### Measures measured repeatedly across sessions.

*Class experience questionnaire:* After each class, participants reflected on their experiences by answering seven questions on a scale ranging from 1 (not at all) to 10 (extremely). The first three questions required participants to rate the class’s challenge level, how destabilizing it was, and their comfort during the session. Subsequently, they reported on the level of support received from both the teacher and their peers and their level of connection with fellow participants. The questionnaire also investigated participants’ level of engagement with the activities proposed during the class.

Finally, the Affect Grid [AG; 54] was used to investigate student emotional experience. The AG comprises the Feeling Scale [FS; 55] and the Felt Arousal Scale [FAS; 56]. The FS is a single-item mood scale ranging from −5 (very bad) to +5 (very good). The FAS is also a single-item scale measuring participants’ arousal, ranging from 1 (low arousal) to 6 (high arousal).

### Intervention

The course was designed by three creative dance teachers and incorporated as a compulsory subject in the second semester of the first year within a Sport and Physical Activity Sciences degree program. The name of the course was “Corporal Expression and Dance”, and it combined creative dance with an introduction to different dance and theatre techniques. It comprised 36 sessions, each lasting 90 minutes, spread over 15 weeks. As designated in Table 1, the course presented three types of classes: movement creativity, theoretical, and presentation.

**Table 1:** Sessions’ schedule of the intervention and class type.

Lessons	Content	Class type
1	Testing (pre-intervention) + Presentation of the subject using practical and creative tasks	Movement
2	Students’ presentation using oral communication + creation of a *Hai Ku*	Theoretical
3	Theory of creativity	Theoretical
4	Group tasks to develop group confidence and creativity	Movement
5	Gestures (improvisation tasks and creation of a video with hand movements) – EB	Movement
**6**	Walking task + representation of personalities by means of the walk – EB	Movement
7 8	Group tasks using rhythm with basket balls to develop confidence Explanation of the evaluation and discussion	Movement Theoretical
9-10-11	Body percussion (creative tasks and creation of rhythm sequences) – EMT	Movement
12-13	Preparation of a performance using body percussion (no creative involvement from students)	Movement
14	Center of mass movements (improvisation using individual and contact tasks) – EB	Movement
15	Individual posture and creation of collective figures – EB	Movement
16-17	Movement in the space (improvisation and creative tasks) – EME	Movement
18	Revision of student’s group choreographies + testing (time 1)	Presentation
19	Energy and qualities of movement (improvisation and creative tasks) – EMQ	Movement
20	Evaluation of the students’ group choreographies	Presentation
21	Energy and qualities of movement (improvisation and creative tasks) – EMQ	Movement
22	Body alignment in dance (no creative involvement from students)	Movement
23-24	Contemporary dance	Movement
25-26	Hip Hop dance (no creative involvement from students)	Movement
27-28-29	Stage combat and fight acting (practice of techniques and creation of sequences)	Movement
30	Production and direction	Theoretical
31	Composing in dance or salsa dance	Movement
32-33-34	Choreographies revision	Presentation
35	Students’ creative oral presentations	Presentation
36	Introduction to circus arts + Testing (post-intervention)	Movement

Most sessions were dedicated to movement creativity (n = 26). They included expressive body movements (EB), spatial movement exploration (EME), and investigations into movement’s relation to time and rhythm (EMT), as well as energy or quality (EMQ). The curriculum also included various dance and theatre techniques, such as contemporary, hip-hop, salsa, and stage combat. One session specifically addressed dance alignment principles. Students were consistently encouraged to innovate by creating new movements, devising novel movement combinations, improvising dance sequences, and applying foundational concepts of physical theatre or creative dance.

Additionally, students engaged in creating and presenting artistic works, such as short dance routines or creative videos emphasizing hand gestures or facial expressions. The concluding session introduced circus techniques, expanding the course’s breadth of creative physical expression. Each session began with an activity to enhance focus and cultivate a creative atmosphere. Opportunities for theoretical discussion or feedback were provided throughout or at the end of each class.

Theoretical sessions (n = 4) were designed to discuss concepts related to expression, creativity development, or the production of an artistic event. One session was focused on explaining the course evaluation criteria. These sessions did not involve creative movements.

The program also featured public presentations of students’ creative projects to the University community or the class (n = 6). After the 13th session, students collaborated on a dance piece addressing sports and gender themes. Following the 34th session, they presented group choreographies at the Artistic Night event, showcasing their creative projects. These presentations, developed with instructor guidance during tutorials and refined in later sessions, formed part of the course assessment. Finally, in the 35th session, students presented a group oral presentation on creative dance’s applicability in various settings, such as educational, sporting, artistic, or wellness contexts.

### Data analysis

A two-step approach was used to determine how participants’ experiences during creative movement classes were associated with changes in study variables. The first stage involved estimating a mixed effect location scale (MELS) model [57]. The MELS is an extension of a traditional mixed effect model, which, unlike a standard model that assumes homogenous errors across all subjects and observations, allows within-subject error variance to vary across participants and time [57]. For the current study, the MELS included the study week and class type as time-varying covariates, group, participant sex, and age as time-varying covariates, and a linear relationship between the random intercept and random scale. The first stage model for participant i at time point j, for outcome y can be seen in the equation below:


$$\begin{array}{cc} y_{ij}= & \;\beta_{\text{0}\text{0}}+\;\gamma_{\text{1}\text{0}}\left({Week}_{ij}\right)+\gamma_{\text{2}\text{0}}\left({Class\;type}_{ij}\right)+\;\gamma_{\text{0}\text{1}}\left({Group}_{i}\right)+\gamma_{\text{0}\text{2}}\left({Sex}_{i}\right)+\;\gamma_{\text{0}\text{3}}\left({Age}_{i}\right)+\;\upsilon_{ij}+\;\varepsilon_{ij}\;\\ & +\;\sigma_{\upsilon ,\varepsilon },\;\upsilon_{ij}\;\sim \;N\left(\text{0},\;\sigma_{\epsilon ij}^{\text{2}}\right),\;\upsilon_{ij}\;\sim \;N\left(\text{0},\;\sigma_{\upsilon ij}^{\text{2}}\right) \end{array}$$

The MELS models the between-subject variances using log-normal models [58]; therefore, within-subject and between-subject variances are estimated as follows:


$$\sigma_{\upsilon ij}^{\text{2}}=\text{exp}(\alpha_{\text{0}})$$

$$\sigma_{\epsilon ij}^{\text{2}}=\text{exp}\left(\tau_{\text{0}}+\omega_{i}\right),\;\omega_{i}\;\sim \;N(\text{0},\sigma_{\omega }^{\text{2}})$$

where α₀ and τ₀ + ωᵢ are the natural logarithms of the between-subject (i.e., random intercept) and within-subject variances, respectively, with ωᵢ allowing the within-subject variance to vary across individuals (i.e., random scale). A separate model was estimated for each indicator of participant experience in class (e.g., challenge, teacher support, affect).

The random intercept (location) and random scale estimated in the first model are subsequently used in the second model as independent variables. The second model is a simple linear regression model. In the current study, the outcome for the second stage model was the study outcomes post-intervention. Each model was controlled for baseline levels of outcomes, participant group, participant age, and participant sex. Therefore, the stage two model was estimated as:


$$y_{i}=\;\beta_{\text{0}}+\;\beta_{\text{1}}\left({\hat{\alpha }}_{\text{0}i}\right)+\;\beta_{\text{2}}\left({\hat{\omega }}_{i}\right)+\;\beta_{\text{3}}\left({Group}_{i}\right)+\;\beta_{\text{4}}\left({Sex}_{i}\right)+\;\beta_{\text{5}}\left({Age}_{i}\right)+\;\beta_{\text{6}}\left({baseline\;y}_{i}\right)+\varepsilon_{i}$$

Because the random intercept (α) and random scale (ω) are estimated values, 1000 resampled datasets were created for each analysis, which were attributed to assuming a normal distribution using the mean and variance estimates. A positive relationship between a random intercept and outcome in the stage 2 model indicates that participants who reported higher values on these variables throughout the intervention had greater increases in outcomes from baseline. A positive relationship between the random scale and outcome indicates that participants who experienced greater variability in variables throughout the intervention had greater increases in the outcome from baseline.

All analyses were conducted using MixWILD [59], and significance was set at p < .05.

The choice of a mixed-effects location scale model (MELS) was driven by its suitability for analyzing complex, hierarchical data structures and capturing variability at multiple levels. Specifically, this model allowed us to address the nested structure of the data, where repeated measures were nested within individuals who were further situated within class groups. This approach was essential to account for between-student and within-student variability in developing creativity-related variables and estimate fixed effects of key predictors (e.g., challenge, comfort, and destabilization) while controlling for random effects such as individual differences and group-level factors. By modeling variability in both location (mean effects) and scale (variability effects), the MELS framework enabled a nuanced exploration of how variability in experiences—such as fluctuations in emotional connection and social support—contributed to creative development.

## Results

### Stage 1 model – Predictors of in-class experiences

Results from the stage 1 model showing factors associated with in-class experiences are displayed in Table 2. In general, results showed that participants reported that movement-based classes were significantly more challenging and destabilizing yet also more comfortable compared to other class types. Additionally, participants reported feeling significantly more supported by the teacher and their peers during movement-based classes than other classes. Participants also felt significantly more engaged and more connected during movement-based classes. Finally, participants reported feeling significantly better mood and high-arousal emotions following movement-based classes than other class types. Participants significantly felt the classes became more challenging, destabilizing, and engaging over the semester, although the increase was minimal. Although statistically significant, these weekly increases were small in magnitude, indicating gradual experiential shifts rather than substantial changes across sessions.

**Table 2:** Regression coefficients (β) from the stage 1 mixed-effects location scale model estimating predictors of in-class experiences and the random scale and location for in-class experiences. The second value next to the regression coefficient represents the standard error (SE). All coefficients are unstandardized.

	Challenge	Destabilizing	Comfort	Support – Teacher	Support – Peer	Engagement	Connection	Arousal	Affect
**Regression coefficients (β)**									
Intercept	3.51 (1.33)**	4.92 (1.42)***	5.74 (0.81)***	7.14 (0.77)***	8.11 (0.79)***	6.07 (0.89)***	7.90 (0.86)***	3.86 (0.61)***	2.49 (0.72)***
Week	0.02 (0.00)***	0.02 (0.00)***	0.01 (0.00)	0.00 (0.00)	−0.01 (0.00)**	0.01 (0.00)*	−0.01 (0.00)	0.01 (0.00)**	0.00 (0.00)
**Class type**									
Movement (ref)									
Theoretical	−0.88 (0.13)***	−0.60 (0.12)***	−0.38 (0.11)***	−0.27 (0.09)**	−0.30 (0.07)***	−0.55 (0.10)***	−0.46 (0.08)***	−0.52 (0.07)***	−0.35 (0.07)***
Presentation	−0.27 (0.12)*	0.09 (0.12)	−0.54 (0.11)***	− 1.31 (0.09)***	−0.17 (0.07)*	−0.31 (0.10) **	−0.13 (0.08)	−0.21 (0.07)**	−0.27 (0.06)***
**Group**									
1 (ref)									
2	−0.38 (0.36)	−0.51 (0.45)	0.59 (0.26)*	0.36 (0.24)	0.42 (0.22)	0.63 (0.28)*	0.40 (0.26)	−0.21 (0.19)	−0.11 (0.20)
3	0.52 (0.38)	0.27 (.46)	0.12 (0.27)	−0.06 (0.25)	−0.03 (0.25)	0.14 (0.29)	−0.07 (0.27)	0.29 (0.20)	0.07 (0.24)
4	0.02 (0.36)	−0.44 (0.42)	0.05 (0.27)	−0.17 (0.21)	−0.08 (0.20)	0.00 (0.28)	−0.22 (0.25)	−0.00 (0.18)	−0.10 (0.15)
**Age**	0.11 (0.05)*	−0.00 (0.06)	0.08 (0.04)*	0.06 (0.04)	0.01 (0.04)	0.07 (0.04)	0.01 (0.04)	0.01 (0.03)	0.03 (0.03)
**Gender**									
Woman (ref)									
Man	−0.03 (0.29)	−0.12 (0.36)	−0.30 (0.21)	−0.27 (0.21)	−0.36 (0.19)	−0.39 (0.23)	−0.36 (0.21)	−0.05 (0.15)	−0.22 (0.19)
**Random intercept (α)**	0.56 (0.16)***	0.93 (0.15)***	−0.06 (0.16)	−0.01 (0.16)	0.03 (0.16)	0.17 (0.16)	0.11 (0.16)	−0.72 (0.16)***	0.07 (0.16)
**Within subject variance (τ)**	1.20 (0.06)***	1.15 (0.07)***	0.93 (0.07)***	0.70 (0.07)***	0.37 (0.09)***	0.86 (0.09)***	0.48 (0.09)***	−0.01 (0.07)	0.44 (0.12)***
**Random scale (ω)**	0.47 (0.05)***	0.60 (0.06)***	0.51 (0.05)***	0.70 (0.07)***	0.70 (0.07)***	0.68 (0.06)***	0.66 (0.06)***	0.54 (0.05)***	0.81 (0.11)***
**Cov(α,τ)**	−0.22 (0.06)***	0.14 (0.08)	−0.30 (0.07)***	−0.39 (0.09)***	−0.59 (0.09)***	−0.40 (0.09)***	−0.49 (0.09)***	−0.20 (0.07)**	−0.23 (0.11)***

* p < .05, ** p < .01, *** p < .001.

Note

Random intercept: Represents the between-subject variability in the baseline values

Random scale: Represents the variability in the effect of the predictors on the outcome across subjects

Gender did not significantly influence the students’ perception of the environment nor their experience. Yet, older participants perceived significantly creative dance as slightly more challenging and comfortable than the younger participants. Also, while most groups experienced creative dance similarly, the second group appeared more comfortable and engaged than the first group.

### Stage 2 model – In-class experiences and changes in intervention outcomes

Results from the stage 2 model examining how in-class experiences are related to changes in intervention outcomes are displayed in Table 3.

**Table 3:** Results from the stage two model regressing intervention outcomes on participant’s random location (i.e., random intercept) and random scale (i.e., within-person variability). The second value next to the regression coefficient represents the standard error (SE). All coefficients are unstandardized.

	CSE	MSTAT	RIBS	ECI – P	ECI – N	ECI – E/A
**Challenge**						
Random intercept (α)	−0.89 (1.54)	−0.04 (0.05)	−0.03 (0.04)	−0.01 (0.07)	0.02 (0.06)	0.10 (0.07)
Random scale (ω)	0.33 (1.71)	−0.05 (0.05)	0.03 (0.05)	−0.04 (0.08)	−0.03 (0.07)	0.10 (0.07)
**Destabilizing**						
Random intercept (α)	5.59 (1.47)***	−0.02 (0.05)	0.01 (0.04)	0.06 (0.07)	0.06 (0.06)	−0.04 (0.06)
Random scale (ω)	−1.50 (1.51)	−0.15 (0.05)**	0.06 (0.04)	−0.07 (0.08)	−0.06 (0.06)	0.03 (0.07)
**Comfort**						
Random intercept (α)	6.03 (1.53)***	0.09 (0.05)	−0.02 (0.04)	0.18 (0.07)*	−0.07 (0.07)	0.19 (0.06)**
Random scale (ω)	−0.96 (1.53)	−0.07 (0.05)	0.07 (0.04)	0.03 (0.07)	−0.02 (0.07)	0.39 (0.07)
**Support – Teacher**						
Random intercept (α)	4.80 (1.55)**	0.10 (0.05)*	−0.03 (0.04)	0.17 (0.07)*	−0.03 (0.07)	0.21 (0.07)**
Random scale (ω)	0.36 (1.53)	−0.10 (0.05)*	0.08 (0.04)	−0.01 (0.08)	0.03 (0.07)	0.02 (0.07)
**Support – Peer**						
Random intercept (α)	5.39 (1.58)***	0.05 (0.04)	−0.02 (0.04)	0.09 (0.08)	−0.04 (0.07)	0.21 (0.07)**
Random scale (ω)	0.42 (1.51)	0.08 (0.05)	0.07 (0.04)	0.08(0.08)	−0.06 (0.06)	0.04 (0.07)
**Engagement**						
Random intercept (α)	7.13 (0.15)***	0.10 (0.05)*	−0.02 (0.04)	0.17 (0.07)*	−0.05 (0.07)	0.19 (0.07)**
Random scale (ω)	−1.14 (1.41)	−0.10 (0.05)*	0.02 (0.04)	0.02 (0.07)	−0.05 (0.06)	0.03 (0.07)
**Connection**						
Random intercept (α)	6.44 (1.54)***	0.06 (0.04)	−0.02 (0.04)	0.11 (0.08)	−0.02 (0.07)	0.20 (0.07)**
Random scale (ω)	−0.71 (1.52)	0.09 (0.05)	0.10 (0.04)*	0.05 (0.08)	−0.02 (0.07)	0.04 (0.07)
**Arousal**						
Random intercept (α)	5.61 (1.47)***	0.05 (0.05)	0.04 (0.04)	0.16 (0.07)*	0.04 (0.03)	0.10 (0.07)
Random scale (ω)	−1.52 (1.50)	−0.11 (0.05)*	0.04 (0.04)	0.07 (0.07)	0.01 (0.07)	0.10 (0.07)
**Affect**						
Random intercept (α)	6.87 (1.64)***	0.07 (0.05)	0.06 (0.04)	0.19 (0.08)*	0.03 (0.07)	0.12 (0.07)
Random scale (ω)	−1.20 (1.50)	−0.06 (0.05)	0.10 (0.04)*	0.05 (0.08)	0.05 (0.06)	0.11 (0.07)

* p < .05, ** p < .01, *** p < .001

Note

Random intercept: Represents the between-subject variability in the baseline values.

Random scale: Represents the variability in the effect of the predictors on the outcome across subjects.

CSE: Creative Self-Efficacy.

MSTAT: Multiple Stimulus Types Ambiguity Tolerance Scale.

RIBS: Runco Ideation Behavior Scale.

ECI-P: Emotional Creativity Inventory – Positive Subscale.

ECI-N: Emotional Creativity Inventory – Negative Subscale.

ECI-E/A: Emotional Creativity Inventory – Emotional/Artistic Subscale.

#### Creative self-efficacy.

Participants who found the classes more destabilizing and comforting over the study period reported a significantly greater increase in creative self-efficacy post-intervention. Additionally, participants who felt more supported by their teacher and peers and felt more connected during the classes also reported significantly greater increases in creative self-efficacy post-intervention. Participants who were more engaged and reported better mood and higher arousal levels following classes also reported significantly greater increases in creative self-efficacy post-intervention. Within-subject variability in class experiences over the study period was not related to creative self-efficacy.

#### Multiple stimulus ambiguity tolerance.

Being more engaged in classes and feeling greater support from the teacher were related to significantly greater improvements in tolerance to ambiguity. On the other hand, experiencing greater variability in teacher support, that is, feeling support in some classes and not others, and engagement, that is, feeling highly engaged in some classes and not engaged in others, was significantly inversely associated with changes in tolerance to ambiguity. Variability in how destabilizing the classes felt was also significantly inversely related to changes in tolerance towards ambiguity.

#### Creative ideational behaviors.

Reporting more positive in-class experiences across the intervention was unrelated to creative ideational behaviors. However, participants who experienced more variability in how much they felt connected, that is, feeling highly connected in some classes and not connected in others, and variability in their mood following classes, that is, feeling well after some classes and not feeling good at all after others, reported significantly greater increases in creative ideational behaviors post-intervention.

#### Emotional creativity – preparedness.

Participants who felt more comfortable and engaged in classes reported significantly greater improvements in their capacity to understand their and other’s emotions, as did participants who felt more support from their teachers during lessons. Participants who reported feeling good or very good mood and feeling more arousal following lessons also reported significantly greater improvements in their ability to understand emotions. Variability in in-class experiences was not related to emotional creativity preparedness.

#### Emotional creativity – novelty.

Neither having more positive in-class experiences nor greater variability in in-class experiences was related to changes in participants’ capacity to experience new or unusual emotions.

#### Emotional creativity – effectiveness/authenticity.

Participants who felt more comfortable and engaged in classes throughout the study reported significantly greater improvements in their ability to express their emotions post-intervention. Additionally, feeling more supported by teachers and peers, and more connected during classes, significantly improved participants’ capacity to express their emotions. Variability in in-class experiences was unrelated to participants’ ability to express their emotions.

## Discussion

This research sought to explore the influence of person-environment interactions on the outcomes of interventions designed to support creative potential development, specifically focusing on students’ experience of creative dance.

The first research question examined how movement creativity classes differed from theoretical and presentation-based classes. In accordance with our first hypothesis (H1), movement creativity classes elicited greater engagement and more positive and high-arousal emotions compared to the other two class types. While students felt significantly more destabilized and connected in movement creativity classes than in theoretical ones, their experiences were similar to those in presentation settings.

The second research question focused on how experiences of challenge, comfort, and destabilization influenced the variables’ development. It was hypothesized that there would be a positive association (H2). The hypothesis was partially confirmed: Exposure to challenge was not significantly associated with the growth of creativity-related variables, but comfort, destabilization, social support and connection did.

The third research question investigated how individual engagement levels and emotional experiences impact the intervention’s outcomes. It was hypothesized that participants reporting greater engagement and positive emotional experiences would benefit more with greater gains in creativity-related outcomes from the intervention (H3). The hypothesis was also confirmed, underscoring the importance of active participation and emotional resonance in maximizing the benefits of creative interventions.

**Fig 1:**
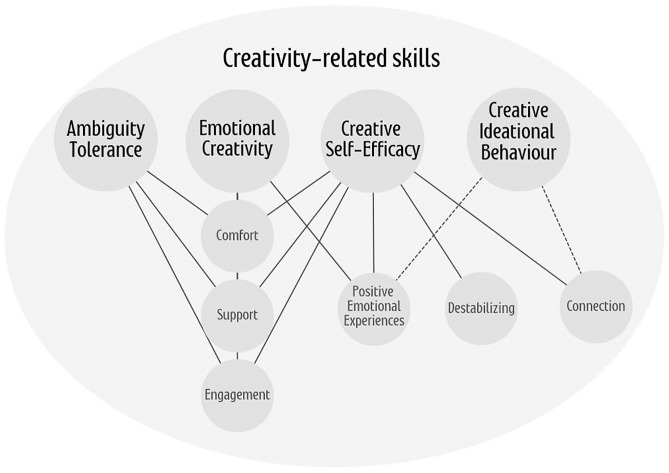
Conceptual model illustrating the relationship between comfort, support, engagement, positive emotions, emotional connection, and creativity outcomes. (-----) Variable experience (_____) Positive association.

Fig 1 summarizes the results, highlighting the fundamental role of comfort, support, and engagement in enhancing most of the creativity-related skills explored in this study. Scholars have previously identified student engagement and social support as key ecological factors in improving learning [60,61], which aligns with the findings presented here. The following section examines these person-environment interactions in greater depth.

### The power of creative embodiment

The enhanced experience perceived by students in creative dance classes may suggest a preference for movement-based activities within the student population of sports sciences. Additionally, the current study findings suggest that embodied approaches may be associated with enhanced learning experiences and the development of creative potential. As Stolz [62] argues, learning processes often neglect the critical role of embodiment and interactions between persons and their environments. Body movement can enhance the study of any discipline or content, providing cognitive benefits and improving knowledge [63–65]. It can also positively impact the classroom environment.

Radical Embodied Cognitive Science (RECS) offers a relevant *post hoc* interpretive tool to make sense of these results. Specifically, RECS proposes that creativity is not just a mental process but is also embodied, embedded, enactive, and expansive (see [66] for details). This perspective suggests that effective creativity training should account for an individual’s physical attributes and how these attributes interact with the surrounding environment. Since aspects such as perception, cognition, emotion, human relations, and behavior are intrinsically linked to our physical existence, exploring physicality as a source of creative development presents a valuable opportunity [67,68]. The current study aligns with the RECS conceptualization of creativity by showing that the creative embodied aspect of creative dance is associated with a more stimulating context to evoke creative growth than other activities that do not incorporate movement or body expression. Creative dance and other creative embodied activities are relatively unexplored [11,69], so more research is needed to examine intervention modalities (e.g., frequency) that lead to creative change and development.

### Rethinking the paradigm: beyond “stepping out of one’s comfort zone”

The impact of creative dance experiences on students’ creative potential development transcends the notion of merely “stepping out of one’s comfort zone”. While feelings of destabilization were notably associated with the development of self-efficacy, the challenge experience did not seem to impact the development of any creativity-related variables. Engagement in creative embodied activities, while also experiencing comfort, support, connection, good mood, and high-arousal emotions, co-occurred with higher levels of creative self-efficacy and emotional creativity in our study population. The magnitude of these associations suggests small-to-moderate practical effects within an educational context.

This perspective aligns with Beghetto and Kaufman’s proposals to nurture creativity, highlighting the importance of establishing a creativity-supportive classroom learning environment [31]. They recommend that teachers integrate creativity into their daily teaching practices, offering opportunities for choice, imagination, and exploration while paying attention to the motivational cues conveyed by classroom practices. Other authors, such as Amabile et al. [70], identified that organizational encouragement, supervisory encouragement, and workgroup encouragement are critical components in promoting creativity within organizations. They suggest encouraging risk-taking, fair and supportive evaluation of new ideas, and collaborative idea flow across an organization significantly contribute to creative outcomes.

Our findings suggest that developmental benefits associated with destabilization may depend on the concurrent presence of supportive and emotionally safe conditions that enable individuals to integrate challenge into adaptive change.

### Emotionally supportive environment

Dance education transcends mere movement outcomes by enhancing both social and emotional competencies [71]. Our findings reinforce this holistic perspective. Students who reported heightened positive emotions and social support during creative dance classes were also more likely to report improvements in emotional understanding. Likewise, students who reported a deeper connection with their peers and social support reported enhanced capabilities in expressing their emotions (effectiveness/authenticity).

Given that socio-cultural norms stringently dictate the expression of emotions within specific contexts [72], the established rapport among participants likely facilitated the public expression of emotions during classes, thereby elucidating these favorable outcomes. Cultural norms surrounding body expression, physical proximity, and nonverbal communication likely shape participants’ comfort or discomfort with movement-based practices. These norms may moderate emotional responses to creative dance, particularly in settings where somatic expression is less culturally valued or embedded in educational practice. We argue that learning to move creatively is a particularly vulnerable and emotionally charged experience, requiring a specific socio-cultural environment.

In the context of creative dance, sociocultural factors, such as the norms of collaboration, shared emotional expression, and supportive and respectful group dynamics, can enhance or constrain the development of creativity-related skills. Consider a scenario in which a student is met with ridicule after exploring different movement possibilities to lyrical music. Such a negative social response could lead to future disengagement from similar activities and environments. Conversely, imagine a student hesitant to move in a certain way but feeling encouraged by peers who take risks first and respect their individual pace of engagement. While this support may not immediately lead the student to actively engage with the environment, it fosters an understanding that such an opportunity is available when they feel ready.

### The paradox of variability in experience

Different patterns of results emerged in how interactions between students and the creative dance environment influenced their development of tolerance for ambiguity and creative ideation behaviors.

Specifically, consistent experiences of engagement and teacher support were positively associated with the development of ambiguity tolerance. However, variability in these experiences and inconsistent feelings of destabilization were associated with negative outcomes. To expand tolerance to ambiguous situations, one must start by consistently exposing oneself to such conditions. This aligns with the idea that to better adapt to stressful situations, one must progressively expose oneself to increased stress levels [e.g., 73]. Yet, the environment must be highly supportive to stimulate positive adaptation to this progressive exposure [74]. Our results are consistent with these ideas. The current intervention aimed to disrupt students’ habitual movement patterns, which might have been perceived as destabilizing and ambiguous for many students. Yet, the creative dance teachers also instilled a stable and supportive environment, striving for an optimal balance between instability (destabilizing movement tasks) and stability (stable, supportive environments). This enables individuals to confront complex and unfamiliar situations effectively. Maintaining stability in other aspects of the environment ensures students can navigate these challenges comfortably and securely.

While the consistency of experience fostered students’ tolerance for ambiguity, the variability of the creative dance experience seemed to stimulate creative ideational behaviors. Specifically, students who reported increased creative ideational behaviors were those who experienced more variability in peers’ connection and better mood after each class. Team members often face disagreements, debates, and occasional frustrations during the choreographic process leading up to the final presentation. However, as the process unfolds, they typically devise unexpectedly creative solutions and reach a consensus, likely facilitated by the process’s cooperative and competitive (i.e., competition between ideas and proposals) nature. These emotional fluctuations and connections with peers may be linked to aspects of the ideational process. For instance, Baas et al. [75] showed that emotions that emerge from appraisals of uncertainty lead to more structured ideation than emotions associated with certainty appraisals. Over time, these creative challenges (aligned with the students’ interests and motivations) may contribute to the emergence of team synergies, enabling flexible interaction among members and ultimately enhancing individual creative ideational behavior [27].

These findings suggest that multiple sociocultural settings can stimulate ideational behaviors. However, the results also highlight the significance of stability in certain aspects, such as teacher support, in fostering tolerance for ambiguity. Collectively, these results suggest that a balance between social stability and variability may be important for supporting creative processes.

#### Practical applications.

The findings of this study hold significant implications for educators and policymakers aiming to foster creativity in learning environments. A key recommendation is to prioritize the establishment of psychologically safe spaces where students feel comfortable taking risks and engaging in creative exploration. Creating such environments requires educators to balance challenge and support, emphasizing positive social connections and collaborative practices. For instance, teachers can design activities encouraging teamwork and peer interaction while modeling openness, empathy, and a nonjudgmental attitude. Integrating embodied approaches like creative dance into curricula provides a powerful avenue for enhancing creativity by engaging students holistically—cognitively, emotionally, and socially. The program included very different proposals related to creative dance, from explorative sessions focused on rhythm, emotional expression, the expressive use of the space or the movement quality to the performance or the experience of some dance techniques, such as hip hop or contemporary dance. The observed effects may reflect more general mechanisms related to motor creativity development, embodied engagement, and affective experience rather than responses to a single, specific activity format.

To support implementation, we suggest several practical strategies: [1] start with activities specifically designed to build emotional safety and mutual trust to foster confidence within the group and towards the teacher; [2] follow up with low-stakes, open-ended tasks that encourage exploration without fear of failure; [3] vary task constraints to introduce challenges through novelty and surprise while maintaining a supportive environment; [4] use group improvisations to enhance social synergy and collective risk-taking; [5] facilitate regular reflection to assist students in interpreting moments of discomfort as part of the creative process; and [6] include opportunities to share creative productions—formally or informally—to reinforce group cohesion, validate individual contributions, and celebrate the creative journey.

Policymakers should consider supporting professional development programs that equip educators with the skills to nurture creativity-supportive environments and invest in resources to integrate embodied and creative practices into educational frameworks. These efforts can help bridge the gap between theoretical insights and practical implementation, ensuring that creativity development is integral to education systems.

### Limitations and future research

The limitations of this study include the characteristics of the sample, as its generalizability may be restricted to this specific population and context. The generalisability to other disciplines (e.g., non movement-based courses) is an open question to be tested in future work. Although participation in the study was voluntary, the creative dance course was mandatory, which could also affect its generalizability. The primary focus of this study was to examine the impact of participants’ experiences of creative dance on creativity-related variables. Therefore, a traditional control group was not included. While the findings highlight the program’s potential benefits, caution is advised when interpreting these results without a comparative reference point. Future research on the influence of person-environment interactions on intervention outcomes should question how best to create a relevant control group with such a design. In light of the results, future studies should also explore how sociocultural factors, such as group norms, cultural expectations, and collective values, influence creative embodied interventions like creative dance. The potential for cultural variability in creative development underscores the importance of tailoring interventions to specific sociocultural contexts to maximize their impact. This would deepen our understanding of the interaction between individuals and their environments and provide actionable insights for educators and policymakers in diverse cultural settings. Additionally, exploring the effects of creative dance on older populations and specific groups focused on wellness, such as individuals in rehabilitation or mental health programs, could further clarify how creativity-based interventions can improve well-being and quality of life. Finally, although efforts were made to develop tailored measurement tools, these tools are subject to subjective interpretations. Future studies should consider using other objective tools to assess creativity development.

## Conclusion

Evidence suggests that training develops individuals’ creative potential [69]. However, interventions have yielded only modest improvements in specific creative dimensions, such as attitude and behavior [76,77]. Our research highlights that interactions between individuals and their environments may play a critical role in shaping outcomes of creative embodied interventions. In a nutshell, how participants experience an intervention matters. We recommend future studies adopt a nuanced person-environment model over a simplistic person-centered approach. This shift could elucidate the mechanisms of creative development [78], informing the design of more effective interventions. For example, creating a supportive environment is critical to nurturing creative potential, emphasizing care, valuing ideas, appreciating the effort, incorporating humor, and demonstrating creativity and passion. Showcasing work, like community performances, connects students and leads to positive outcomes. While theoretical accounts highlight the potential benefits of challenge, our findings did not support the notion that discomfort or disequilibrium independently promotes creativity. Instead, emotional safety and a supportive atmosphere appeared to be more closely associated with students’ creative engagement in this context. It’s important to note that our participants had no prior experience in dance, and individuals with greater familiarity or expertise might benefit differently from creative tensions or higher levels of challenge. Yet, these strategies are seldom applied in Physical Education or traditional dance education, which often prioritize replication over creative movement and emotions, neglecting self-awareness and emotional expression [79–81]. While the manifold benefits of dance practice and Physical Education are widely acknowledged, their potential could be further enhanced by integrating creativity development within learning methodologies, particularly in a supportive and comfortable environment.
